# Supplementary material for: Effects of Water Restriction and Supplementation on Cognitive Performances and Mood among Young Adults in Baoding, China: A Randomized Controlled Trial (RCT)
Source: Nutrients. 2021 Oct 18;13(10):3645. doi: 10.3390/nu13103645 (PMC8539979; doi:10.3390/nu13103645)
Supplement: Supplementary file 1 [file nutrients-13-03645-s001.zip › nutrients-1379668-supplementary.pdf]

Table S1 The criteria for the inclusion and exclusion of participants

Inclusion criteria	
	Aged 18-23 years
	Be healthy
Exclusion criteria	
	Aged <18 or >23 years
	Tobacco use
	Habitual alcohol (>20 g/day) consumption
	Habitual high caffeine (>250 mg/day) consumption
	Vigorous-intensity physical activity habits
	Chronic diseases
	Claustrophobia
	Other neurologic illness
	Other medical illness

Table S2 The outcomes of the study

The scores of the CP using software after intervention (primary outcome)
The scores of the POMS using questionnaire after intervention (primary outcome)
Urine osmolality, USG and the electrolytes concentrations
Plasma osmolality, the electrolytes concentrations and glucose
Anthropometric measurements including the height, weight and blood pressure

CP: cognitive performance; POMS: Profile of mood states; USG: Urine specific gravity
